# Spatiotemporal Patterns of CRF07_BC in China: A Population-Based Study of the HIV Strain With the Highest Infection Rates

**DOI:** 10.3389/fimmu.2022.824178

**Published:** 2022-02-14

**Authors:** Mengze Gan, Shan Zheng, Jingjing Hao, Yuhua Ruan, Lingjie Liao, Yiming Shao, Yi Feng, Hui Xing

**Affiliations:** State Key Laboratory of Infectious Disease Prevention and Control, National Center for AIDS/STD Control and Prevention (NCAIDS), Chinese Center for Disease Control and Prevention (China CDC), Collaborative Innovation Center for Diagnosis and Treatment of Infectious Diseases, Beijing, China

**Keywords:** HIV, CRF07_BC, diffusion trajectory, molecular network, phylogeographic analysis

## Abstract

The prevalence of CRF07_BC is 39.7% and has become the most infectious HIV strain in China. To study the transmission and diffusion trajectory of CRF07_BC in China and to prevent further expansion of its transmission. A total of 16,635 sequences of the CRF07_BC *pol* gene were collected from 1997-2020. We characterized the gene subtypes according to a phylogenetic tree analysis. A 0.50% molecular network was constructed to analyze the transmission relationship among different provinces for CRF07_BC and its two epidemic clusters. Spatial and temporal propagation characteristics were analyzed according to phylogeographic analysis. Finally, we evaluated the differences in transmission of CRF07_BC-O, and CRF07_BC-N. Our dataset included 8,816 sequences of CRF07_BC-N and 7,819 sequences of CRF07_BC-O. There were 7,132 CRF07_BC sequences in the molecular network, and the rate of clustered was 42.9%. Compared to CRF07_BC-O, CRF07_BC-N showed significantly (*P*<0.001) higher transmission-specific rates. CRF07_BC originated among injecting drug users (IDUs), and spread to men who have sex with men (MSMs) and heterosexual individuals (HETs), while MSMs also transmitted directly to HETs. CRF07_BC-O and CRF07_BC-N were prevalent in Xinjiang and Sichuan, respectively, before spreading interprovincially. In modern China, CRF07_BC-N occurs in five of the major economic zones. The CRF07_BC strain, which has contributed to the highest number of HIV infections in China, is divided into two epidemic clusters. Compared with CRF07_BC-O, risk of transmission is much greater in CRF07_BC-N, which is predominantly prevalent in economically developed provinces, and both MSMs and IDUs have transmitted this epidemic cluster to HETs. High-resolution, large-scale monitoring is a useful tool in assessing the trend and spread of the HIV epidemic. The rapidly developing economy of China requires an equally rapid response to the prevention and control of infectious diseases.

## Introduction

CRF07_BC was initially characterized among HIV positive among injecting drug users (IDUs) in 1996 and is one of the most predominant Circulating Recombinant Forms (CRFs) of HIV strains in China (1–3). The HIV subtype B' strain from Thailand spread to Yunnan around 1985 (4). In 1989, the HIV subtype C strain from India spread to Yunnan (5–7). In the same year, an AIDS epidemic was declared among IDUs in Dehong, Yunnan province (8). CRF07_BC was formed by recombination of subtype B' from Thailand and subtype C from India and has spread to many provinces in less than 30 years. Two epidemic clusters of CRF07_BC have since been identified in China (9). CRF07_BC-O was genetically and epidemiologically similar to the original CRF07_BC, circulating in IDUs and heterosexual individuals (HETs). It was also called CRF07_BC-unclustered or CRF07_BC-Others in some studies (9–11). CRF07_BC-N is the latest cluster derived from CRF07_BC-O and has mostly been reported in men who have sex with men (MSM). It has also been called CRF07_BC-Cluster 1 or CRF07_BC-MSM in some studies (9–11).

CRF07_BC has become the most infectious HIV strain in China; the latest data from the China CDC indicated that the prevalence of CRF07_BC is 39.7% (11, 12). To better prevent and control the transmission of this strain, it is necessary to grasp the demographic characteristics, geographical distribution, and transmission patterns of the different virus strains, especially for the two lesser studied epidemic clusters of CRF07_BC-N and CRF07_BC-O. Therefore, this study focuses on the spatial and temporal transmission characteristics of CRF07_BC and its two epidemic clusters.

## Materials and Methods

### Study Population

A total of 20,202 sequences of the CRF07_BC *pol* gene region (HXB2: 2,253-3,312 nt, 1,060 bp), dating between 1997 and 2020, were sourced from the HIV sequence databases of the Los Alamos National Laboratories (https://www.hiv.lanl.gov/, accessed Jun 30, 2020) and the National Center for AIDS/STD Control and Prevention (Beijing, China). We used HXB2 as a reference sequence for sequences alignment using MAFFT. FastTree v2.1.11 and RaxmlGUI v2.0.0 were used in phylogenetic tree construction to determine gene subtypes and subclusters. The sequence inclusion criteria were: length ≥1,000 bp, mixed base <5%, and the availability of province and sampling year data. Duplicated sequences were removed. The final dataset consisted of 16,635 sequences of CRF07_BC covering all 31 provinces and major cities of China. This study was approved by an ethics committee (Ethics Committee of National Center for AIDS/STD Control and Prevention, Chinese CDC, X140617334).

### Phylogenetic Analysis and HIV Molecular Network Construction

A molecular network was constructed using HIV-TRACE (Transmission Cluster Engine) (13). We aligned HIV *pol* sequences to an HXB2 reference sequence and calculated pairwise genetic distances under the Tamura-Nei 93 model (14). The ambiguous nucleotides of all sequences were less than 5% and used RYWSMK for handling mixed bases (recommended by software developers). Each node in the molecular network represented an individual, and was linked to another node if their pairwise genetic distance was up to 0.50% substitutions per site (We wanted to understand the spread of CRF07_BC in recent years and referred to the guidelines of the Centers for Disease Control and Prevention, USA, so we chose a genetic distance of 0.05%) (15).

Using the molecular network, we evaluated the differences in transmission between two CRF07_BC epidemic clusters in China by the rate of clustered, network density (ND), and rate of inter-provincial transmission (RIPT), respectively.


$$\begin{array}{c} Clustered(\%)=\frac{The\;number\;of\;individuals\;entering\;the\;molecules\;network}{The\;total\;number\;of\;Individual}\\ ND=\frac{2L}{N(n-1)} \end{array}$$

N represents the node and L is the ratio of the actual number of edges in the network to the upper limit of edges that can be accommodated. The ND describes the density and concurrency level of the network.


$$\mathrm{RIPT}\;(\%)=\frac{\mathrm{The}\;\mathrm{number}\;\mathrm{of}\;\mathrm{individuals}\;\mathrm{positive}\;\mathrm{for}\;\mathrm{interprovincial}\;\mathrm{transmission}}{\mathrm{The}\;\mathrm{total}\;\mathrm{number}\;\mathrm{of}\;\mathrm{individuals}\;\mathrm{in}\;\mathrm{the}\;\mathrm{province}}$$

Inter-provincial transmission is defined as the relatedness of the sequence in the molecular network to those in different provinces, confirming that the corresponding HIV/AIDS strain has been transmitted inter-provincially. Intra-provincial transmission is defined as the relatedness of the sequence in the molecular network to those in the same province, confirming that the corresponding HIV/AIDS strain has been transmitted intra-provincially.

Inter-provincial transmission rate is easily affected by sample size: small sample size can simulate a high inter-provincial transmission rate. Therefore, we adjusted our analyses to reflect simultaneous inter-provincial and intra-provincial transmission (SIT) as well as the rate of simultaneous inter-provincial and intra-provincial transmission (RSIT). 


SIT (%)=Intra− provincial transmission∩inter−provincial transmission

$$\mathrm{RSIT}\;(\%)=\frac{\mathrm{The}\;\mathrm{number}\;\mathrm{of}\;\mathrm{individuals}\;\mathrm{positive}\;\mathrm{for}\;\mathrm{SIT}}{\mathrm{The}\;\mathrm{total}\;\mathrm{number}\;\mathrm{of}\;\mathrm{individuals}\;\mathrm{in}\;\mathrm{the}\;\mathrm{province}}$$

This method indicated that simultaneous inter-provincial and intra-provincial transmission is an important determinant for province-specific infection rates and can help us identify the target population for disease control and prevention. Hot spots of inter-provincial transmission were defined as those with RSIT above the national overall level. Provinces with simultaneous inter-provincial transmission and intra-provincial transmission <10 sequences/individual were not included in the analyses.

### Time-Scaled Phylogenetic Tree Reconstruction Using BEAST

A sub-sample was selected from the original dataset for Bayesian analysis. Criteria for the sub-sample dataset: (1) the distribution of subtypes and subclusters was estimated and the sample size of each province was determined based on annual HIV/AIDS statistics and the results of four national surveys; (2) Random sampling, referring to the transmission route (risk), sex, age and other components of infected people in each province, while ensuring the distribution of sampling year in each province; (3) at least 10 sequences from each province were selected for analysis. The final dataset consisted of 500 sequences for Bayesian analysis. The subsampling procedure was repeated multiple times, though the results were equivalent.

To evaluate the temporal and spatial dynamics of CRF07_BC in various provinces, we performed a Bayesian discrete phylogeographic approach to estimate the rate of evolution and the time to the most recent common ancestor (tMRCA). The analysis consisted of 200 million Markov chain Monte Carlo (MCMC) iterations using BEAST v1.8.4 (16) under a Bayesian Skygrid demographic model (17). The final dataset was analyzed using a general time-reversible (GTR) nucleotide substitution model (gamma distribution prior on each relative substitution rate) and a relaxed uncorrelated lognormal (UCLN) molecular clock model to infer the timescale of HIV-1 evolution (gamma distribution prior on the mean clock rate) (18, 19). The Bayesian MCMC output was analyzed using Tracer v1.6 (20). The Effective Sample Size (ESS) values for each estimate was >200. The first 10-30% of states from each run were discarded as burn-in. Trees were summarized by maximum clade credibility (MCC) using TreeAnnotator and then visualized in FigTree v1.4.4. SpreaD3 v0.9.7.1 (21) was used to draw the CRF07_BC propagation roadmap (22).

### Discrete Phylogeographic Analyses and Spatial Structure

To provide an adequate description of the process of viral dissemination, we used a Bayesian stochastic search variable selection (BSSVS) (23) procedure. We aimed to analyze the relationship between transmission risk groups (Risk), risk and sex (Risk-Sex), and risk and age (Risk-Age). We also used a robust counting (Markov jumps) (24) approach to estimate the expected number of virus lineage movements. The level of statistical support was estimated using Bayes factors (BF)23 and summarized using SpreaD3.

### Statistical Analysis

The chi-square test was used to calculate the differences of the two epidemic clusters in the molecular network, CRF07_BC-N and CRF07_BC-O. A Wilcoxon rank-sum test was used to estimate regional differences between five economic zones. P<0.05 was statistically significant. All statistical tests were performed using R v4.0.2.

## Results

### Prevalence of the Two CRF07_BC Epidemic Clusters

By phylogenetic tree analysis (See reference 9 for the phylogenetic trees), we identified that the dataset consisted of 53.0% (8,816/16,635) CRF07_BC-N sequences and 47.0% (7,819/16,635) CRF07_BC-O sequences (**Table 1**). In CRF07_BC-N, 58.6% (5,168/8,816) were male, 28.4% (2,507/8,816) were 18-29 years old, 46.4% (4,089/8,816) were MSMs, and 15.3% (1,345/8,816) were HETs. In CRF07_BC-O, 58.6% (5,168/7,819) were male, 25.2% (1,969/7,819) were 18-29 years old, 46.7% (3,655/7,819) were HETs, and 25.8% (2,017/7,819) were IDUs. CRF07_BC-O and CRF07_BC-N had statistically significant differences in risk, sex and provinces distribution, see **Supplementary Table S1**.

**Table 1:** Demographic characteristics of CRF07_BC and the two epidemic clusters.

		Overall	Risk				Sampling year	Province (n)
			MSM	HET	IDU	Others		
CRF07_BC	North	2989	1041	272	76	1600	2004-2019	BJ (2325), TJ (41), HE (387), SX (173), NM (63)
	Northeast	307	225	51	6	25	2000-2019	LN (113), JL (99), HLJ (95)
	East	2458	1182	717	66	493	2004-2020	SH (491), JS (459), ZJ (349), AH (620), FJ (87), JX (210), SD (242)
	Center	790	335	273	9	173	2005-2019	HA (225), HB (177), HN (388)
	South	2909	763	388	99	1659	2006-2019	GD (1639), SZ (803), GX (423), HI (44)
	Southwest	5415	591	2576	1561	687	1997-2019	CQ (730), SC (907), LS (2853), GZ (369), YN (518), XZ (38)
	Northwest	1747	238	723	228	578	1997-2019	SN (305), GS (118), QH (34), NX (70), XJ (1240)
	Overall	16635	4375	5000	2045	5215	1997-2019	31 provinces
CRF07_BC-N	North	2458	973	192	4	1289	2004-2019	BJ (1881), TJ (35), HE (334), SX (147), NM (61)
	Northeast	253	205	30	1	17	2007-2019	LN (89), JL (81), HLJ (83)
	East	1716	1119	285	6	306	2007-2020	SH (451), JS (320), ZJ (216), AH (439), FJ (75), JX (51), SD (164)
	Center	629	317	172	1	139	2008-2019	HA (197), HB (141), HN (291)
	South	2121	740	211	4	1166	2006-2019	GD (1105), SZ (717), GX (262), HI (37)
	Southwest	1204	540	342	10	312	2007-2019	CQ (544), SC (205), LS (21), GZ (246), YN (176), XZ (12)
	Northwest	435	195	113	2	125	2006-2019	SN (244), GS (88), QH (26), NX (46), XJ (31)
	Overall	8816	4089	1345	28	3354	2004-2019	31 provinces
CRF07_BC-O	North	531	68	80	72	311	2005-2019	BJ (444), TJ (6), HE (53), SX (26), NM (2)
	Northeast	54	20	21	5	8	2000-2019	LN (24), JL (18), HLJ (12)
	East	742	63	432	60	187	2004-2020	SH (40), JS (139), ZJ (133), AH (181), FJ (12), JX (159), SD (78)
	Center	161	18	101	8	34	2005-2019	HA (28), HB (36), HN (97)
	South	788	23	177	95	493	2006-2019	GD (534), SZ (86), GX (161), HI (7)
	Southwest	4211	51	2234	1551	375	1997-2019	CQ (186), SC (702), LS (2832), GZ (123), YN (342), XZ (26)
	Northwest	1332	43	610	226	453	1997-2019	SN (61), GS (30), QH (8), NX (24), XJ (1209)
	Overall	7819	286	3655	2017	1861	1997-2019	31 provinces

Others include mother-to-child transmission, blood transfusion transmission and unknown. Shenzhen and Liangshan were listed separately due to the large number of sequences collected. Guangdong was not included in the sequences of Shenzhen, and Sichuan was not included in the sequences of Liangshan. Risk: HET, heterosexual; MSM, men who have sex with men; IDU, injecting drug users. Provinces: Anhui (AH), Beijing (BJ), Chongqing (CQ), Fujian (FJ), Guangdong (GD), Gansu (GS), Guangxi Zhuang Autonomous Region (GX), Guizhou (GZ), Henan (HA), Hubei (HB), Hebei (HE), Hainan (HI), Heilongjiang (HLJ), Hunan (HN), Jilin (JL), Jiangsu (JS), Jiangxi (JX), Liaoning (LN), Liangshan (LS), Inner Mongolia Autonomous Region (NM), Ningxia Hui Autonomous Region (NX), Qinghai (QH), Sichuan (SC), Shandong (SD), Shanghai (SH), Shaanxi (SN), Shanxi (SX), Shenzhen (SZ), Tianjin (TJ), Xinjiang Uygur Autonomous Region (XJ), Tibet Autonomous Region (XZ), Yunnan (YN), Zhejiang (ZJ).

### Molecular Network Analysis of CRF07_BC and the Two Epidemic Clusters

In the constructed molecular network, 7,132 sequences were clustered. CRF07_BC was 42.9% (7,132/16,635) clustered. CRF07_BC-N (59.1%, 5,211/8,816) was significantly more clustered (χ2=2018.46, P<0.001) than CRF07_BC-O (24.6%, 1,921/7,819). The ND of CRF07_BC-N (6.19×10-3) was 4.7 times that of CRF07_BC-O (1.31×10-3) and the RIPT of CRF07_BC-N (44.7%, 3,938/8,816) was also significantly higher (χ2=2889.80, P<0.001) than that of CRF07_BC-O (7.5%, 587/7,819). The RSIT of CRF07_BC-N (28.2%, 2,489/8,816) was significantly higher (χ2=1956.28, P<0.001) than that of CRF07_BC-O (2.9%, 223/7,819).

**Figure 1:**
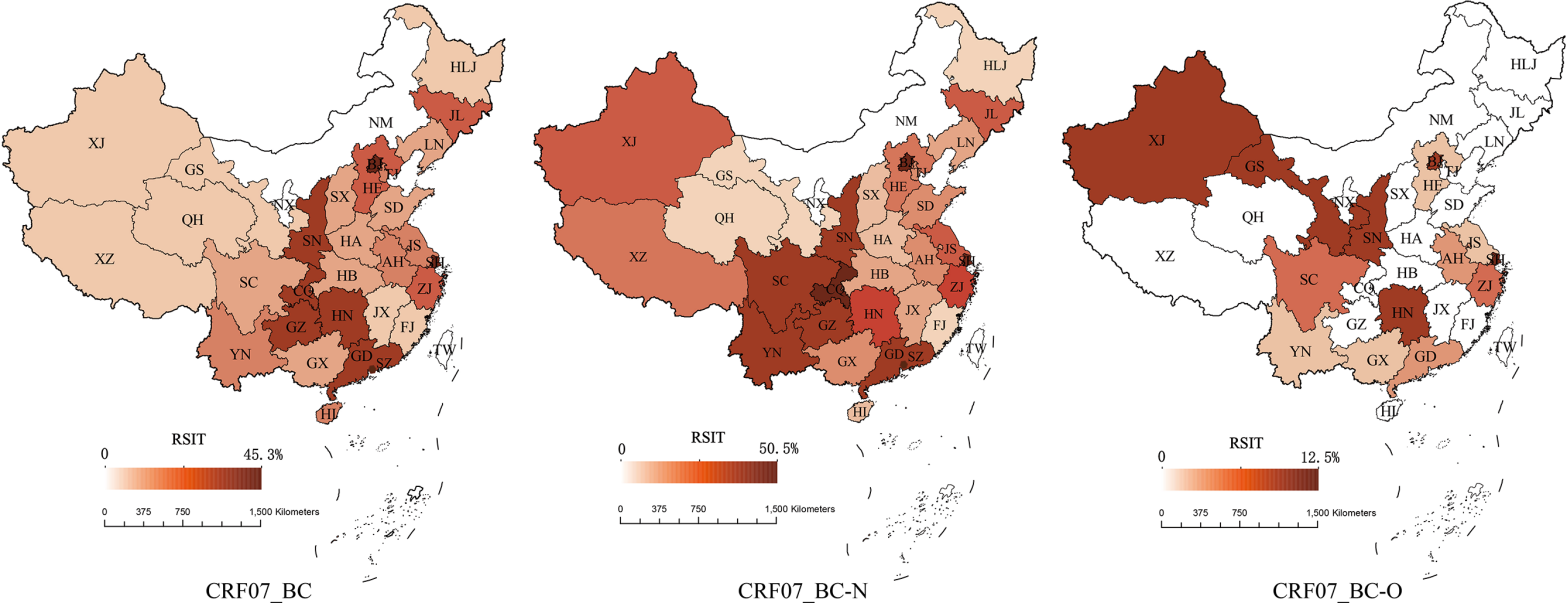
The RSIT distribution of CRF07_BC, CRF07_BC-N, and CRF07_BC-O. The redder the color, the higher the RSIT. White provinces indicate no SIT.

The inter-provincial transmission of CRF07_BC exceeded intra-provincial transmission, and nine provinces had RSITs above the national overall level. The CRF07_BC hot spots were SZ, SH, BJ, CQ, SN, HN, GZ, GD, and ZJ. CRF07_BC-N showed higher inter-provincial transmission compared to intra-provincial transmission, and there were nine provinces with RSIT above the national overall level. The hot spots of CRF07_BC-N were SZ, SH, CQ, BJ, GZ, YN, GD, SN, and SC. The inter-provincial transmission of CRF07_BC-O was higher than the intra-provincial transmission, and there were nine provinces with RSIT above the national overall level. The hot spots of CRF07_BC-O were SH, GS, SN, HN, BJ, XJ, ZJ, and SC (**Figure 1** and **Supplementary Tables S2**–**S4**).

### Analysis of the Temporal and Spatial Transmission Characteristics of CRF07_BC and the Two Epidemic Clusters

**Figure 2:**
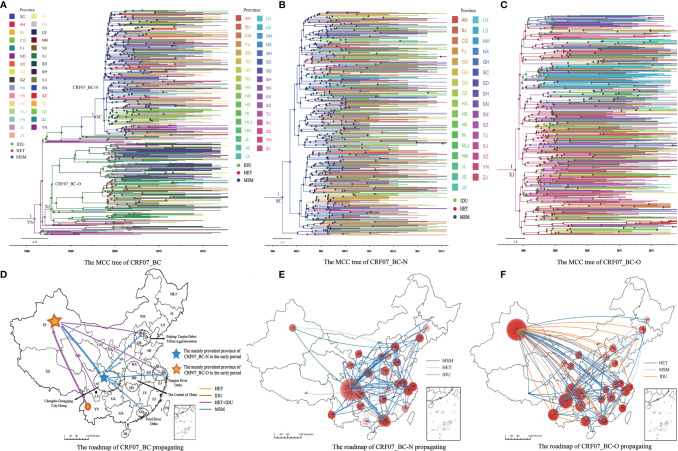
In the MCC tree, the color of the branch indicates the most probable ancestral region. The number on the branch represents posterior probability. The color of the node indicates the transmission route (risk). In the propagation roadmap, the lines in the figure show the posterior probability ≥ 0.8. The color of the lines represents risk. The red circles indicate the absolute and relative intensity of local spread. **(A)** CRF07_BC originated in Yunnan and formed two clusters during its transmission, CRF07_BC-O cluster in Xinjiang and CRF07_BC-N cluster in Sichuan. **(B)** This is the MCC tree of CRF07_BC-N, which is mainly prevalent in Sichuan. **(C)** This is the MCC tree of CRF07_BC-O, which is mainly prevalent in Xinjiang. **(D)** As CRF07_BC-N is a particularly prevalent strain of HIV in China, its main transmission and epidemic regions are depicted in the figure by a black dotted line. **(E)** This is the propagation roadmap of CRF07_BC-N, which is mainly prevalent in Sichuan. **(F)** This is the propagation roadmap of CRF07_BC-O, which is mainly prevalent in Xinjiang.

We reconstructed the epidemiological history of CRF07_BC in China. The results showed that the tMRCA of CRF07_BC was in 1995.9, YN (95% confidence interval (CI): 1994.5-1997.6; posterior probability=1). In early 1997.7 (95% CI: 1997.0-1997.7) it was mainly prevalent in XJ and was transmitted from IDUs to HETs. It then spread to LS (in the southwest of SC), LN, and other provinces, forming the CRF07_BC-O cluster. In addition, after spreading to MSMs in SC, CRF07_BC was mainly prevalent in Chengdu (the capital city of SC province) in 2003.7 (95% CI: 2002.4-2004.8), then spread to CQ, GD, SN, and other provinces, forming the CRF07_BC-N cluster. After 2005, CRF07_BC and the two epidemic clusters increased rapidly. After 2010, CRF07_BC-N prevalence exceeded that of CRF07_BC-O (**Figure 2** and **Supplementary Figures S1, S2**).

### Inferring Mixture of CRF07_BC-N Transmission Behaviors

The molecular network analysis indicated a higher risk of transmission in CRF07_BC-N compared to CRF07_BC-O. Therefore, we focused our analyses on the transmission of CRF07_BC-N.

**Figure 3:**
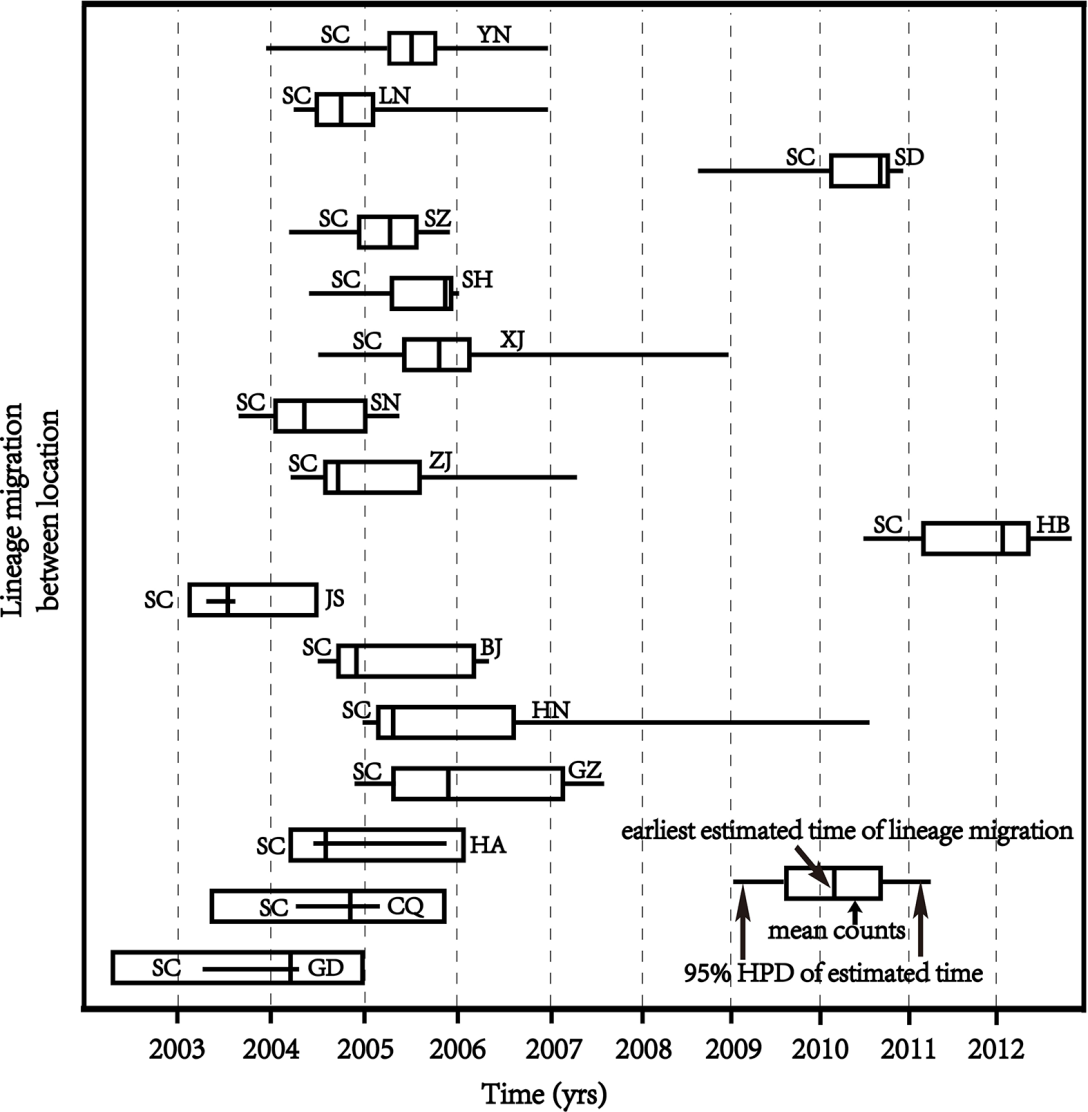
CRF07_BC-N spread to various provinces after the outbreak in SC. The figure shows the 16 provinces with a related strain to SC (posterior probability ≥0.8). The length of the box represents the mean counts. The horizontal line represents the transmission time and 95% confidence interval from point A to point B The vertical line corresponds to the earliest transmission time from point A to point B.

**Figure 4:**
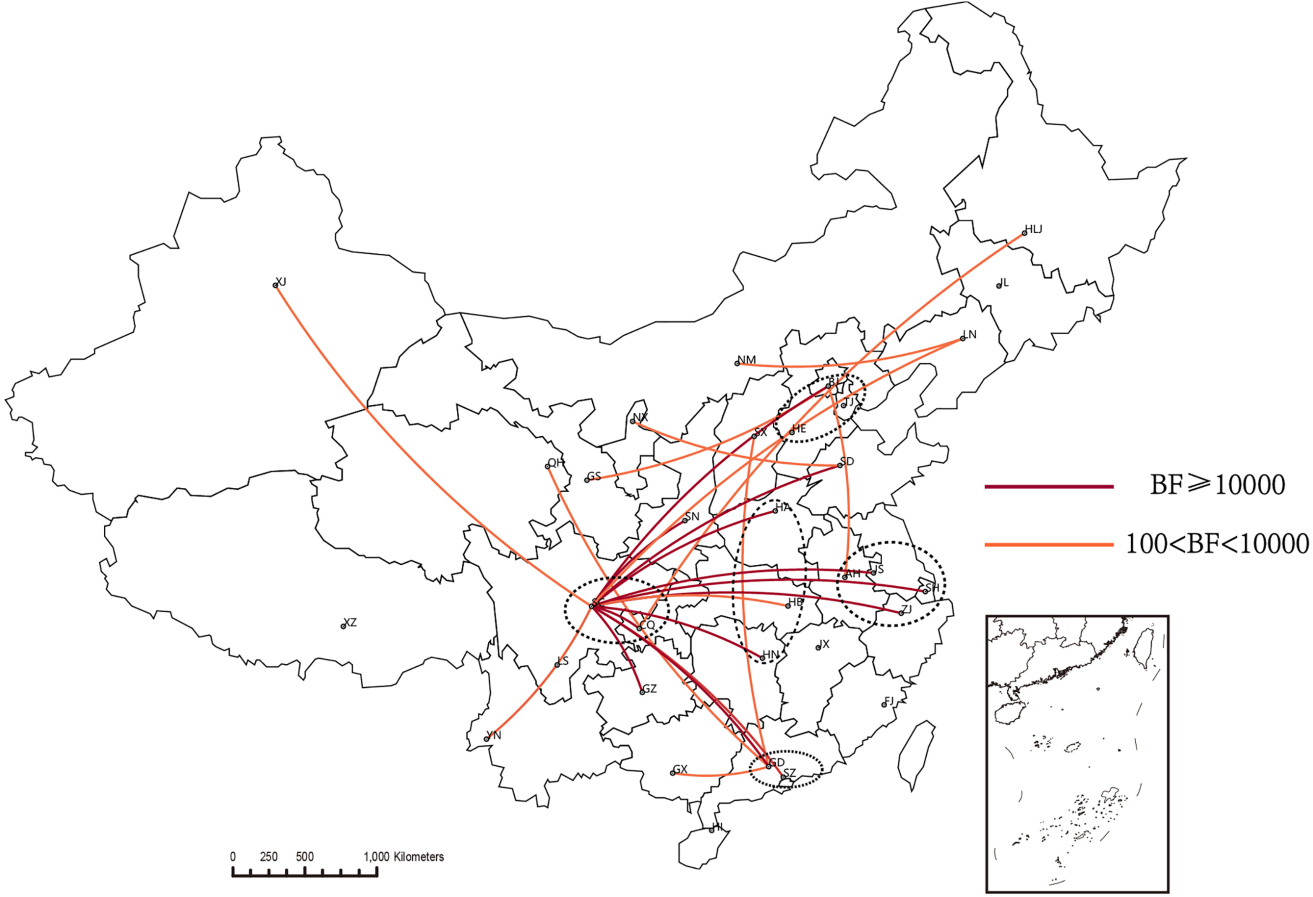
The map of key transmission provinces of CRF07_BC-N. The transmission relationships of provinces with posterior probability ≥0.8 are shown in the figure. The main transmission relationships in the figure were BF>10,000.

The BSSVS analysis found that CRF07_BC-N mainly spread among 25 provinces, involving seven regions, including SC, GD, CQ, HA, GZ, HN, BJ, JS, ZJ, SN, SH, SZ, SD, YN, HB, XJ, LN, GS, SX, HLJ, AH, GX, QH, NX, NM (BF ≥100, posterior probability ≥0.8). Sixteen provinces were broadcast from SC, also involving 7 regions, namely GD, CQ, HA, GZ, HN, BJ, JS, HB, ZJ, SN, XJ, SH, SZ, SD, LN, and YN (BF ≥100, posterior probability ≥0.8). See **Table 2** and **Figures 3**, **4** for details.

**Table 2:** BSSVS results of CRF07_BC-N in the provinces.

From	To	Mean counts	Bayes Factor	Posterior probability^*^
SC	GD	81.49	>10000	1.00
SC	CQ	75.87	>10000	1.00
SC	HA	56.39	>10000	1.00
SC	GZ	55.28	>10000	1.00
SC	HN	44.20	>10000	1.00
SC	BJ	44.18	>10000	1.00
SC	JS	41.39	>10000	1.00
SC	HB	34.27	5404.92	0.99
SC	ZJ	30.73	>10000	1.00
SC	SN	29.27	>10000	1.00
SC	XJ	21.25	426.76	0.93
SC	SH	19.33	>10000	1.00
SC	SZ	18.70	>10000	1.00
SC	SD	18.45	>10000	1.00
SC	LN	18.35	907.20	0.97
SC	YN	15.01	>10000	1.00
BJ	GS	6.28	269.45	0.90
GD	SX	5.29	1693.97	0.98
CQ	HLJ	2.90	208.00	0.87
BJ	AH	2.83	235.23	0.88
GD	GX	2.46	180.40	0.85
GD	QH	2.45	135.24	0.81
LN	NM	1.25	170.46	0.84
SD	NX	1.08	339.99	0.92

*The transmission relationships with posterior probability ≥0.8 were selected.

**Figure 5:**
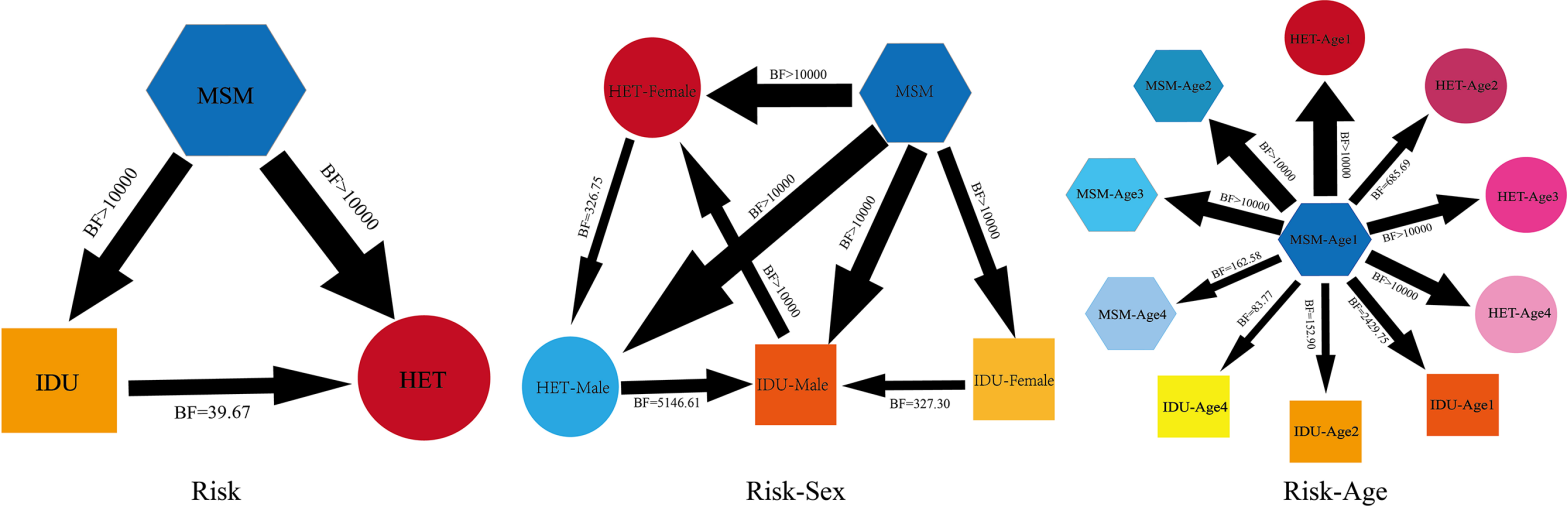
The transmission relationships among different populations in CRF07_BC-N. The thickness of the arrows in the figure indicates the magnitude of BF and posterior probability. Risk-Sexs were classified into HET-Female (females in heterosexuals), HET-Male (males in heterosexuals), IDU-Male (males in injecting drug users), and MSM. Age1: 18-29 years old, Age2: 30-39 years old, Age3: 39-49 years old, Age4: ≥50 years old.

Transitions between Risk with the highest statistical support were from MSMs to HETs (BF>10,000, posterior probability=1) and IDUs (BF>10,000, posterior probability=1). Transitions between Risk-Sex with the highest support were from MSMs to HETs-Male (BF>10,000, posterior probability=1), HETs-Female (BF>10,000, posterior probability=1), and IDUs-Male (BF>10,000, posterior probability=1). Transitions between Risk-Age with the highest support were from MSMs aged 18-29 to HETs and IDUs aged 18-29 (posterior probability=1) (**Figure 5** and **Supplementary Tables S5**–**S7**). CRF07_BC-O transmission behaviors are shown in **Supplementary Tables S8**–**S10**.

### Correlation Analysis Between the Spread of CRF07_BC-N and Five Economic Zones

**Figure 6:**
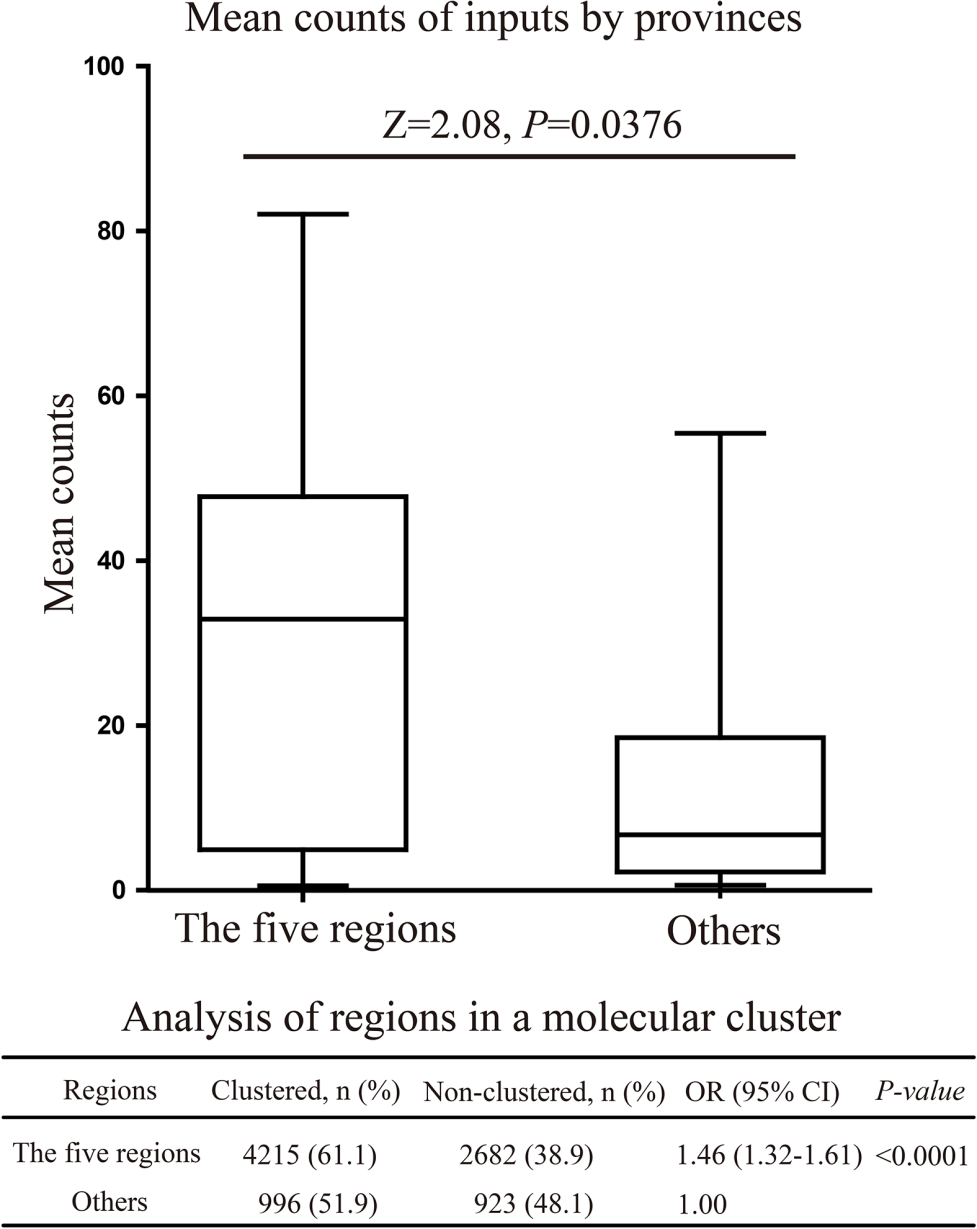
Correlation analysis between CRF07_BC-N of the five economic zones.

CRF07_BC-N was mainly transmitted from SC to 13 provinces (BF>10,000, posterior probability=1) (**Figure 4**). The above provinces are located in several important economic zones, including the Beijing-Tianjin-Hebei Urban Agglomeration, the Yangtze River Delta, the Pearl River Delta, the Chengdu-Chongqing City Group, and Central China. Simultaneous analysis comparing the five economic zones with the other regions was carried out using molecular network and Bayesian correlation analyses. CRF07_BC-N was found to be more easily accessible to the molecular network in the five economic zones (OR=1.46, range=1.32-1.61, P<0.0001). Comparing the mean counts of provinces in the five economic zones with other regions, the results showed that provinces with greater CRF07_BC-N prevalence were more likely to be located in the five economic zones (P=0.0376, **Figure 6**).

## Discussion

We used a nationwide sequence database spanning from 1997-2020 to systematically analyze the origin and spatial and temporal transmission characteristics of CRF07_BC and its epidemic clusters, CRF07_BC-N and CRF07_BC-O. CRF07_BC was identified approximately 25 years ago. The formation of CRF07_BC-N was later than that of CRF07_BC-O, but its transmission rates were much higher. By 2020, it has been identified in all provinces of China and has exceeded the prevalence of CRF07_BC-O (53.0% of CRF07_BC-N, 47.0% of CRF07_BC-O). The analysis results showed that the differences between CRF07_BC-O and CRF07_BC-N in risk, sex, and provinces were statistically significant, suggesting that they were differences of the transmission in demographic characteristics. Transmission risk and pattern also varied between these epidemic clusters.

Through BSSVS analysis, we characterized the main epidemic populations of CRF07_BC-N and CRF07_BC-O. There was mutual transmission among various populations in the two epidemic clusters, reflecting the complex interactive transmission modes among HIV infected individuals in China. HETs males were the bridging population in the transmission between MSMs and HETs. This could be related to a high proportion of undisclosed MSMs in China. MSMs in China may have both homosexual and heterosexual behaviors at the same time. Therefore, it is necessary to strengthen the intervention of MSMs.

Four transmission indicators (the rate of clustered, ND, RIPT, and RSIT) were analyzed using a molecular network. Bayesian analysis and the molecular network analysis showed that the transmission risk of CRF07_BC-N was higher than that of CRF07_BC-O. CRF07_BC-N is widely spread across China, and may be related to the movements of people due to economic, cultural, and technological reasons. The epidemic cluster is mainly transmitted among MSM, with increased transmission to HETs. The importance of the MSM population to transmission may be related to etiology. For example, studies have shown high transmission efficiency of subtype B through MSM and IDUs, while the transmission efficiency of subtype A, subtype C, and CRF01_AE is high specifically in HETs (25, 26). Differences in transmission between subtypes of the strains may be due to differences in the ability of these strains to invade Langerhans cells in the mucosa of the reproductive tract (27). Therefore, further analysis of the pathogenic characteristics of the clusters is required, and CRF07_BC-N requires special attention in the prevention and control of its transmission to the larger population.

After the rapid spread of CRF07_BC-N in Sichuan province, it successfully spread to five economic zones (**Figure 2D**). The concept of economic circles was introduced in the 1990s and eight subsequent economic development circles were established. Each region within the economic circle stimulates the growth of the other, and China's economy developed rapidly as a result. AIDS has spread to China mainly through sexual transmission. The spread of HIV was therefore strongly associated with the large-scale population fluctuations caused by China’s rapid economic development. These five economic zones include the most attractive provinces and cities with regards to transportation, culture, and scientific development. CRF07_BC-N has spread predominantly among MSM in large and medium-sized cities. Therefore, the spread of CRF07_BC-N in these five economic zones has been increasingly affected by social development.

We recognize that certain limitations exist in our study. There is a potential for variation in the collection of HIV gene sequences. For provinces with rapid progress in HIV molecular epidemiology, more HIV gene sequences need to be obtained. For provinces where the progress of HIV molecular epidemiology is relatively slow, the availability of HIV gene sequences is limited. The provinces labeled in the sequence data relate to the location where the sample was collected and might not reflect the province of origin, making inter-provincial transmission analysis precarious.

This study found that the spread of CRF07_BC-N in China was closely related to the large-scale movement of people caused by the rapid development of the economy and transportation. Therefore, the detailed and timely epidemiological characterization of the different HIV strains could help society prevent transmission and control the severity of the epidemic through molecular surveillance. At the same time, given the influence of an expanding economy and continuous innovation in transportation, it is important to establish adaptive frameworks for the prevention and control of infectious diseases as soon as possible.

## Data Availability Statement

The datasets presented in this study can be found in online repositories. The names of the repository/repositories and accession number(s) can be found in the article/**Supplementary Material**.

## Ethics Statement

The studies involving human participants were reviewed and approved by Ethics Committee of National Center for AIDS/STD Control and Prevention, Chinese CDC, X140617334. The patients/participants provided their written informed consent to participate in this study.

## Author Contributions

MG was responsible for the study design, analysis, and writing. SZ was responsible for data verification, statistics, and figures. JH was responsible for data verification, statistics, and tables. YR, LL, and YS were responsible for revising the article. YF and HX were responsible for guiding the whole study and revising the article. All authors have read and approved the final version of the manuscript.

## Funding

This study was funded by the Ministry of Science and Technology of China (Grant number: 2017ZX10201101002-004), Beijing Municipal Science and Technology Commission (Grant number: D161100000416002), The fund providers had no role in the study design, data collection and analysis, decision to publish, or preparation of the manuscript.

## Conflict of Interest

The authors declare that the research was conducted in the absence of any commercial or financial relationships that could be construed as a potential conflict of interest.

## Publisher’s Note

All claims expressed in this article are solely those of the authors and do not necessarily represent those of their affiliated organizations, or those of the publisher, the editors and the reviewers. Any product that may be evaluated in this article, or claim that may be made by its manufacturer, is not guaranteed or endorsed by the publisher.
