# Supplementary material for: Spatiotemporal Patterns of CRF07_BC in China: A Population-Based Study of the HIV Strain With the Highest Infection Rates
Source: Front Immunol. 2022 Feb 14;13:824178. doi: 10.3389/fimmu.2022.824178 (PMC8882613; doi:10.3389/fimmu.2022.824178)
Supplement: Supplementary file 1 [file DataSheet_1.docx]

Supplementary Material

# Supplementary Figures and Tables

**Table S1.** Demographic characteristics comparison between CRF07_BC-O and CRF07_BC-N

|  | CRF07_BC-O | CRF07_BC-N | χ2 | *P*-value |
| --- | --- | --- | --- | --- |
| Overall | 7819 | 8816 |  |  |
| Risk |  |  | 6699.28 | <0.0001 |
| MSM | 286 | 4089 |  |  |
| HET | 3655 | 1345 |  |  |
| IDU | 2017 | 28 |  |  |
| Others | 1861 | 3354 |  |  |
| Sex |  |  | 2280.77 | <0.0001 |
| Male | 3604 | 5168 |  |  |
| Female | 2274 | 238 |  |  |
| Unknown | 1941 | 3410 |  |  |
| Province |  |  | 7640.45 | <0.0001 |
| AH | 181 | 439 |  |  |
| BJ | 444 | 1881 |  |  |
| CQ | 186 | 544 |  |  |
| FJ | 12 | 75 |  |  |
| GD | 534 | 1105 |  |  |
| GS | 30 | 88 |  |  |
| GX | 161 | 262 |  |  |
| GZ | 123 | 246 |  |  |
| HA | 28 | 197 |  |  |
| HB | 36 | 141 |  |  |
| HE | 53 | 334 |  |  |
| HI | 7 | 37 |  |  |
| HLJ | 12 | 83 |  |  |
| HN | 97 | 291 |  |  |
| JL | 18 | 81 |  |  |
| JS | 139 | 320 |  |  |
| JX | 159 | 51 |  |  |
| LN | 24 | 89 |  |  |
| LS | 2832 | 21 |  |  |
| NM | 2 | 59 |  |  |
| NX | 24 | 48 |  |  |
| QH | 8 | 26 |  |  |
| SC | 702 | 205 |  |  |
| SD | 78 | 164 |  |  |
| SH | 40 | 451 |  |  |
| SN | 61 | 244 |  |  |
| SX | 26 | 147 |  |  |
| SZ | 86 | 717 |  |  |
| TJ | 6 | 35 |  |  |
| XJ | 1209 | 31 |  |  |
| XZ | 26 | 12 |  |  |
| YN | 342 | 176 |  |  |
| ZJ | 133 | 216 |  |  |

**Table S2.** Provincial distribution of CRF07_BC in the molecular network

| Province | Overall (%) | Clustered (%) | RIPT (%) | RSIT (%) |
| --- | --- | --- | --- | --- |
| Overall | 16635 (100.0) | 7132 (42.9) | 4525 (27.2) | 2716 (16.3) |
| **SZ** | **803 (4.8)** | **515 (64.1)** | **434 (54.0)** | **364 (45.3)** |
| **SH** | **491 (3.0)** | **315 (64.2)** | **270 (55.0)** | **188 (38.3)** |
| **BJ** | **2325 (14.0)** | **1378 (59.3)** | **975 (41.9)** | **678 (29.2)** |
| **CQ** | **730 (4.4)** | **346 (47.4)** | **242 (33.2)** | **189 (25.9)** |
| **SN** | **305 (1.8)** | **144 (47.2)** | **111 (36.4)** | **74 (24.3)** |
| **HN** | **388 (2.3)** | **195 (50.3)** | **147 (37.9)** | **84 (21.6)** |
| **GZ** | **369 (2.2)** | **191 (51.8)** | **120 (32.5)** | **78 (21.1)** |
| **GD** | **1639 (9.9)** | **724 (44.2)** | **529 (32.3)** | **336 (20.5)** |
| **ZJ** | **349 (2.1)** | **169 (48.4)** | **118 (33.8)** | **63 (18.1)** |
| JL | 99 (0.6) | 52 (52.5) | 42 (42.4) | 16 (16.2) |
| HE | 387 (2.3) | 183 (47.3) | 133 (34.4) | 61 (15.8) |
| JS | 459 (2.8) | 189 (41.2) | 129 (28.1) | 66 (14.4) |
| AH | 620 (3.7) | 412 (66.5) | 138 (22.3) | 76 (12.3) |
| YN | 518 (3.1) | 180 (34.7) | 117 (22.6) | 61 (11.8) |
| TJ | 43 (0.3) | 26 (60.5) | 24 (55.8) | 5 (11.6) |
| HI | 44 (0.3) | 16 (36.4) | 13 (29.5) | 5 (11.4) |
| SD | 242 (1.5) | 119 (49.2) | 99 (40.9) | 25 (10.3) |
| LN | 113 (0.7) | 48 (42.5) | 43 (38.1) | 11 (9.7) |
| HB | 177 (1.1) | 74 (41.8) | 53 (29.9) | 17 (9.6) |
| GX | 423 (2.5) | 191 (45.2) | 93 (22.0) | 40 (9.5) |
| HA | 225 (1.4) | 69 (30.7) | 65 (28.9) | 21 (9.3) |
| SC | 907 (5.5) | 265 (29.2) | 199 (21.9) | 81 (8.9) |
| SX | 173 (1.0) | 78 (45.1) | 52 (30.1) | 15 (8.7) |
| GS | 118 (0.7) | 41 (34.7) | 28 (23.7) | 8 (6.8) |
| HLJ | 95 (0.6) | 43 (45.3) | 36 (37.9) | 6 (6.3) |
| XJ | 1236 (7.4) | 316 (25.6) | 100 (8.1) | 76 (6.1) |
| FJ | 87 (0.5) | 30 (34.5) | 22 (25.3) | 5 (5.7) |
| XZ | 38 (0.2) | 10 (26.3) | 8 (21.1) | 2 (5.3) |
| JX | 210 (1.3) | 103 (49.0) | 23 (11.0) | 7 (3.3) |
| QH | 34 (0.2) | 14 (41.2) | 8 (23.5) | 1 (2.9) |
| LS | 2853 (17.2) | 646 (22.6) | 125 (4.4) | 57 (2.0) |
| NM | 65 (0.4) | 26 (40.0) | 19 (29.2) | 0 (0.0) |
| NX | 70 (0.4) | 24 (34.3) | 10 (14.3) | 0 (0.0) |

**Table S3.** Provincial distribution of CRF07_BC-N in the molecular network

| Province | Overall (%) | Clustered (%) | RIPT (%) | RSIT (%) |
| --- | --- | --- | --- | --- |
| Overall | 8816 (100.0) | 5211 (59.1) | 3938 (44.7) | 2489 (28.2) |
| **SZ** | **717 (8.1)** | **495 (69.0)** | **420 (58.6)** | **362 (50.5)** |
| **SH** | **451 (5.1)** | **297 (65.9)** | **258 (57.2)** | **183 (40.6)** |
| **CQ** | **544 (6.2)** | **314 (57.7)** | **238 (43.8)** | **189 (34.7)** |
| **BJ** | **1881 (21.3)** | **1240 (65.9)** | **907 (48.2)** | **653 (34.7)** |
| **GZ** | **246 (2.8)** | **152 (61.8)** | **116 (47.2)** | **78 (31.7)** |
| **YN** | **176 (2.0)** | **114 (64.8)** | **92 (52.3)** | **55 (31.3)** |
| **GD** | **1105 (12.5)** | **649 (58.7)** | **489 (44.3)** | **323 (29.2)** |
| **SN** | **244 (2.8)** | **126 (51.6)** | **105 (43.0)** | **70 (28.7)** |
| **SC** | **205 (2.3)** | **126 (61.5)** | **118 (57.6)** | **58 (28.3)** |
| ZJ | 216 (2.5) | 135 (62.5) | 108 (50.0) | 58 (26.9) |
| HN | 291 (3.3) | 167 (57.4) | 134 (46.0) | 78 (26.8) |
| JS | 320 (3.6) | 158 (49.4) | 117 (36.6) | 64 (20.0) |
| JL | 81 (0.9) | 48 (59.3) | 38 (46.9) | 16 (19.8) |
| XJ | 31 (0.4) | 11 (35.5) | 11 (35.5) | 6 (19.4) |
| HE | 334 (3.8) | 171 (51.2) | 124 (37.1) | 60 (18.0) |
| XZ | 12 (0.1) | 5 (41.7) | 5 (41.7) | 2 (16.7) |
| AH | 439 (5.0) | 300 (68.3) | 126 (28.7) | 72 (16.4) |
| SD | 164 (1.9) | 98 (59.8) | 81 (49.4) | 25 (15.2) |
| TJ | 35 (0.4) | 24 (68.6) | 22 (62.9) | 5 (14.3) |
| GX | 262 (3.0) | 142 (54.2) | 82 (31.3) | 37 (14.1) |
| JX | 51 (0.6) | 22 (43.1) | 20 (39.2) | 7 (13.7) |
| LN | 89 (1.0) | 43 (48.3) | 38 (42.7) | 11 (12.4) |
| HB | 141 (1.6) | 66 (46.8) | 50 (35.5) | 17 (12.1) |
| HI | 37 (0.4) | 14 (37.8) | 12 (32.4) | 4 (10.8) |
| HA | 197 (2.2) | 67 (34.0) | 63 (32.0) | 21 (10.7) |
| SX | 147 (1.7) | 75 (51.0) | 50 (34.0) | 15 (10.2) |
| LS | 21 (0.2) | 6 (28.6) | 4 (19.0) | 2 (9.5) |
| HLJ | 83 (0.9) | 38 (45.8) | 33 (39.8) | 6 (7.2) |
| GS | 88 (1.0) | 31 (35.2) | 22 (25.0) | 6 (6.8) |
| FJ | 75 (0.9) | 28 (37.3) | 22 (29.3) | 5 (6.7) |
| QH | 26 (0.3) | 12 (46.2) | 8 (30.8) | 1 (3.8) |
| NM | 61 (0.7) | 24 (39.3) | 18 (29.5) | 0 (0.0) |
| NX | 46 (0.5) | 13 (28.3) | 7 (15.2) | 0 (0.0) |

**Table S4.** Provincial distribution of CRF07_BC-O in the molecular network

| Province | Overall (%) | Clustered (%) | RIPT (%) | RSIT (%) |
| --- | --- | --- | --- | --- |
| Overall | 7819 (100.0) | 1921 (24.6) | 587 (7.5) | 223 (2.9) |
| **HI** | **7 (0.1)** | **2 (28.6)** | **1 (14.3)** | **1 (14.3)** |
| **SH** | **40 (0.5)** | **18 (45.0)** | **12 (30.0)** | **5 (12.5)** |
| **GS** | **30 (0.4)** | **10 (33.3)** | **6 (20.0)** | **2 (6.7)** |
| **SN** | **61 (0.8)** | **13 (21.3)** | **6 (9.8)** | **4 (6.6)** |
| **HN** | **97 (1.2)** | **28 (28.9)** | **13 (13.4)** | **6 (6.2)** |
| **BJ** | **444 (5.7)** | **143 (32.2)** | **68 (15.3)** | **25 (5.6)** |
| **XJ** | **1205 (15.4)** | **302 (25.1)** | **89 (7.4)** | **66 (5.5)** |
| **ZJ** | **133 (1.7)** | **34 (25.6)** | **10 (7.5)** | **5 (3.8)** |
| **SC** | **702 (9.0)** | **139 (19.8)** | **81 (11.5)** | **23 (3.3)** |
| GD | 534 (6.8) | 76 (14.2) | 40 (7.5) | 13 (2.4) |
| SZ | 86 (1.1) | 20 (23.3) | 14 (16.3) | 2 (2.3) |
| AH | 181 (2.3) | 111 (61.3) | 12 (6.6) | 4 (2.2) |
| LS | 2832 (36.2) | 640 (22.6) | 121 (4.3) | 55 (1.9) |
| HE | 53 (0.7) | 11 (20.8) | 9 (17.0) | 1 (1.9) |
| GX | 161 (2.1) | 49 (30.4) | 11 (6.8) | 3 (1.9) |
| YN | 342 (4.4) | 66 (19.3) | 25 (7.3) | 6 (1.8) |
| JS | 139 (1.8) | 31 (22.3) | 12 (8.6) | 2 (1.4) |
| HLJ | 12 (0.2) | 5 (41.7) | 3 (25.0) | 0 (0.0) |
| NM | 4 (0.1) | 3 (75.0) | 1 (25.0) | 0 (0.0) |
| TJ | 8 (0.1) | 2 (25.0) | 2 (25.0) | 0 (0.0) |
| SD | 78 (1.0) | 21 (26.9) | 18 (23.1) | 0 (0.0) |
| JL | 18 (0.2) | 4 (22.2) | 4 (22.2) | 0 (0.0) |
| LN | 24 (0.3) | 5 (20.8) | 5 (20.8) | 0 (0.0) |
| NX | 24 (0.3) | 11 (45.8) | 3 (12.5) | 0 (0.0) |
| XZ | 26 (0.3) | 5 (19.2) | 3 (11.5) | 0 (0.0) |
| HB | 36 (0.5) | 7 (19.4) | 3 (8.3) | 0 (0.0) |
| SX | 26 (0.3) | 5 (19.2) | 2 (7.7) | 0 (0.0) |
| HA | 28 (0.4) | 2 (7.1) | 2 (7.1) | 0 (0.0) |
| GZ | 123 (1.6) | 39 (31.7) | 4 (3.3) | 0 (0.0) |
| CQ | 186 (2.4) | 34 (18.3) | 4 (2.2) | 0 (0.0) |
| JX | 159 (2.0) | 81 (50.9) | 3 (1.9) | 0 (0.0) |
| FJ | 12 (0.2) | 2 (16.7) | 0 (0.0) | 0 (0.0) |
| QH | 8 (0.1) | 2 (25.0) | 0 (0.0) | 0 (0.0) |

In this study, the whole Bayesian analysis of CRF07_BC and the separate Bayesian analysis of CRF07_BC-N and CRF07_BC-O were performed. The aim was to reconstruct the complete epidemic history of the CRF07_BC. The results showed that the tMRCA of CRF07_BC-O in the whole Bayesian of CRF07_BC was 1995.9 (1994.5-1997.6), and that of CRF07_BC-N was 2001.2 (1997.8-2002.4). CRF07_BC-O showed that tMRCA was 1993.8 (1984.7-1995.0), and CRF07_BC-N showed that tMRCA was 2001.0 (2000.7-2003.6), as shown in **Figure S1**.


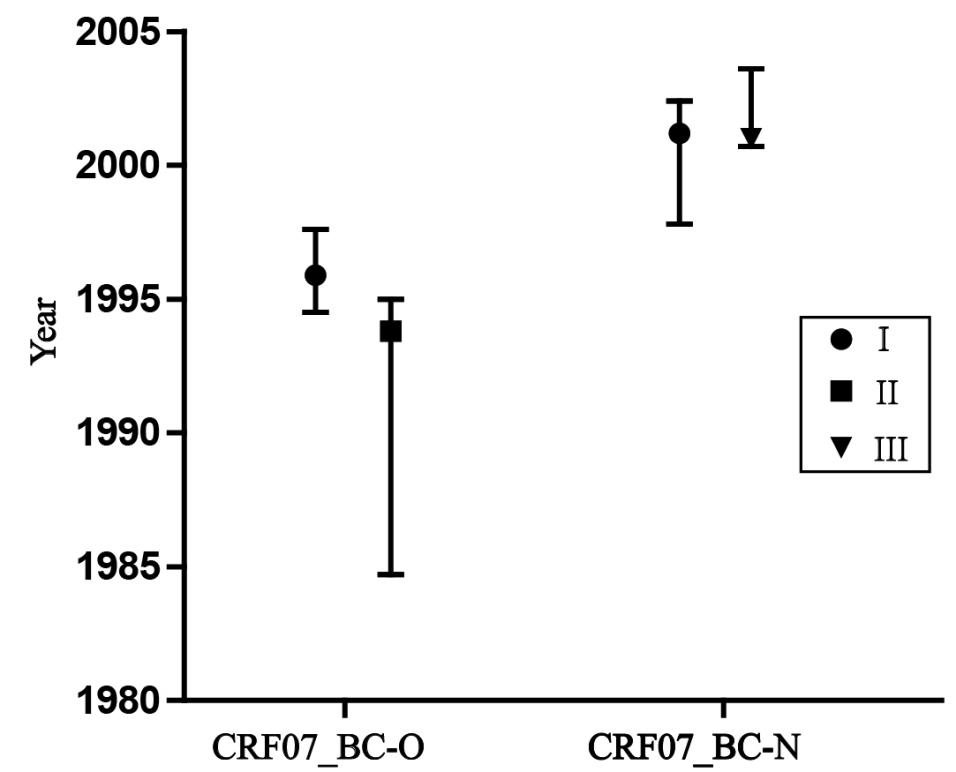


**Figure S1.** The tMRCA of CRF07_BC, CRF07_BC-N, and CRF07_BC-O

Note: I indicates the result of whole Bayesian for CRF07_BC. II indicates the result of CRF07_BC-O alone. III represents the result of CRF07_BC-N alone.

Further, Bayesian Skygrid Plot was used to describe the demographic characteristics of CRF07_BC, CRF07_BC-N and CRF07_BC-O. Bayesian Skygrid Plot can reflect the change trend of effective population size (Ne) over time. Bayesian Skygrid Plot showed that CRF07_BC showed an overall trend of growth. CRF07_BC-N had a rapid growth period from its origin to about 2015. CRF07_BC-O had a rapid growth period from its origin to about 2010, **Figure S2**.


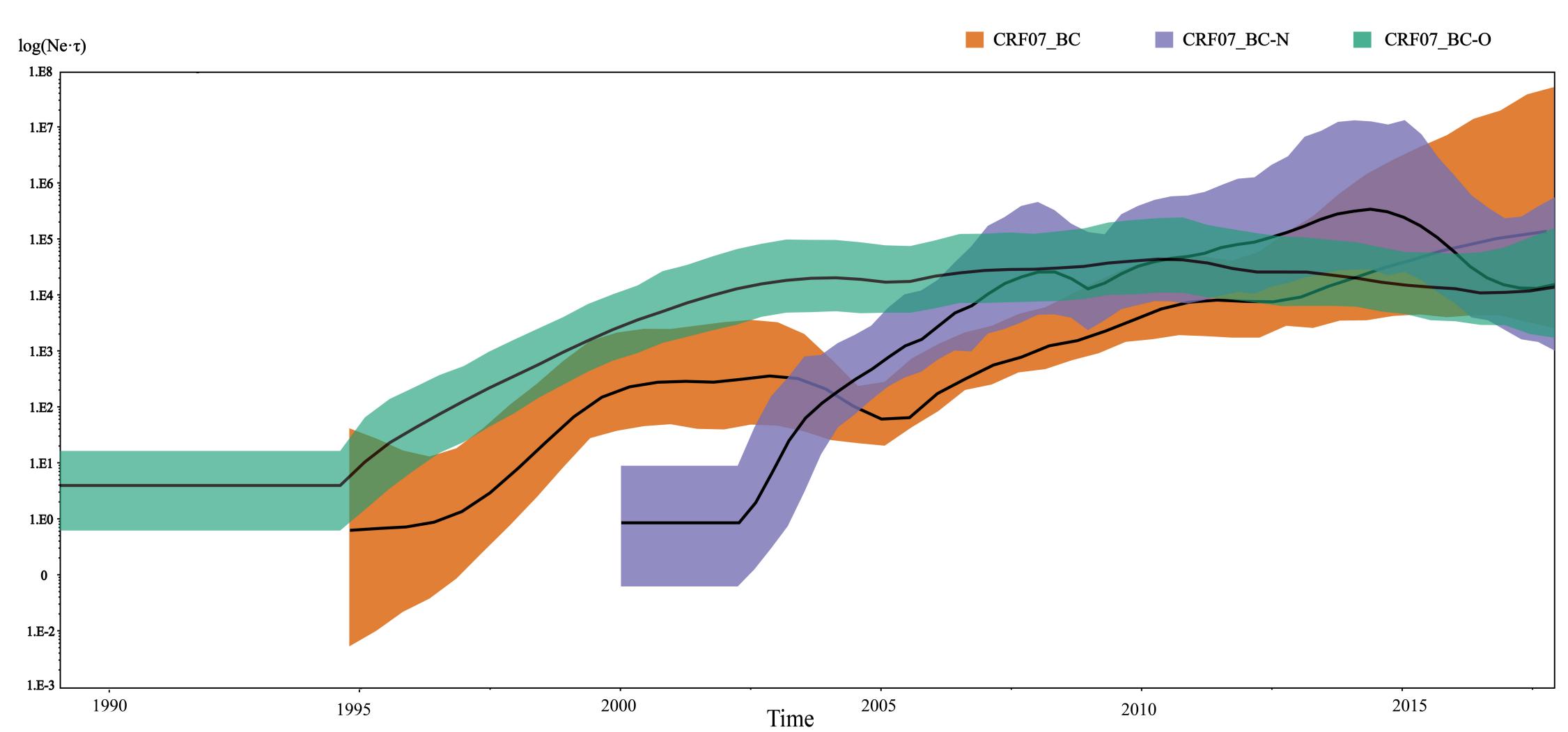


**Figure S2.** Bayesian Skygrid Plot of CRF07_BC, CRF07_BC-N, and CRF07_BC-O

**Table S5.** BSSVS results of CRF07_BC-N in the Risk

| From | To | Mean counts | Bayes Factor | Posterior probability^*^ |
| --- | --- | --- | --- | --- |
| MSM | HET | 111.31 | >10000 | 1.00 |
| MSM | IDU | 25.40 | >10000 | 1.00 |
| IDU | HET | 0.01 | 39.67 | 0.97 |

*: The transmission relationships with posterior probability ≥0.8 were selected. HET, heterosexual. MSM, men who have sex with men. IDU, injecting drug users.

**Table S6.** BSSVS results of CRF07_BC-N in the Risk-Sex

| From | To | Mean counts | Bayes Factor | Posterior probability^*^ |
| --- | --- | --- | --- | --- |
| MSM | HET-Male | 87.46 | >10000 | 1.00 |
| MSM | HET-Female | 26.90 | >10000 | 1.00 |
| MSM | IDU-Male | 22.74 | >10000 | 1.00 |
| MSM | IDU-Female | 3.32 | >10000 | 1.00 |
| HET-Male | IDU-Male | 0.39 | 5146.61 | 1.00 |
| HET-Female | HET-Male | 0.06 | 326.75 | 0.99 |
| IDU-Male | HET-Female | 0.03 | >10000 | 1.00 |
| IDU-Female | IDU-Male | 0.01 | 327.30 | 0.99 |

*: The transmission relationships with posterior probability ≥0.8 were selected. Risk-Sexs were classified into HET-Female (females in heterosexuals), HET-Male (males in heterosexuals), IDU-Male (males in injecting drug users), and MSM.

**Table S7.** BSSVS results of CRF07_BC-N in the Risk-Age

| From | To | Mean counts | Bayes Factor | Posterior probability^*^ |
| --- | --- | --- | --- | --- |
| MSM-Age1 | HET-Age1 | 81.00 | >10000 | 1.00 |
| MSM-Age1 | MSM-Age2 | 43.60 | >10000 | 1.00 |
| MSM-Age1 | MSM-Age3 | 22.76 | >10000 | 1.00 |
| MSM-Age1 | HET-Age4 | 19.34 | >10000 | 1.00 |
| MSM-Age1 | IDU-Age1 | 16.92 | 2429.75 | 1.00 |
| MSM-Age1 | HET-Age2 | 14.92 | 685.69 | 0.99 |
| MSM-Age1 | HET-Age3 | 13.00 | >10000 | 1.00 |
| MSM-Age1 | MSM-Age4 | 12.76 | 162.58 | 0.94 |
| MSM-Age1 | IDU-Age2 | 6.24 | 152.90 | 0.94 |
| MSM-Age1 | IDU-Age4 | 2.97 | 83.77 | 0.89 |

*: The transmission relationships with posterior probability ≥0.8 were selected. Age1: 18-29 years old, Age2: 30-39 years old, Age3: 39-49 years old, Age4: ≥50 years old.

**Table S8.** BSSVS results of CRF07_BC-O in the provinces

| From | To | Mean counts | Bayes Factor | Posterior probability^*^ |
| --- | --- | --- | --- | --- |
| XJ | SC | 94.84 | >10000 | 1.00 |
| XJ | LS | 68.01 | >10000 | 1.00 |
| XJ | GD | 39.53 | >10000 | 1.00 |
| XJ | YN | 37.21 | >10000 | 1.00 |
| XJ | CQ | 33.23 | >10000 | 1.00 |
| XJ | GZ | 31.56 | >10000 | 1.00 |
| XJ | BJ | 28.18 | >10000 | 1.00 |
| XJ | JS | 22.54 | >10000 | 1.00 |
| XJ | ZJ | 21.63 | >10000 | 1.00 |
| XJ | SD | 21.22 | >10000 | 1.00 |
| XJ | GX | 19.23 | >10000 | 1.00 |
| XJ | FJ | 12.51 | 221.84 | 0.88 |
| XJ | LN | 12.11 | >10000 | 1.00 |
| SC | SZ | 5.42 | 205.00 | 0.87 |
| SC | HE | 4.15 | 187.83 | 0.86 |
| SC | TJ | 3.69 | 401.23 | 0.93 |
| LS | SX | 2.65 | 2195.93 | 0.99 |
| LS | JX | 1.75 | 7074.62 | 1.00 |
| GZ | XZ | 1.38 | 421.58 | 0.93 |

*: The transmission relationships with posterior probability ≥0.8 were selected.

**Table S9.** BSSVS results of CRF07_BC-O in the Risk

| From | To | Mean counts | Bayes Factor | Posterior probability^*^ |
| --- | --- | --- | --- | --- |
| IDU | HET | 161.50 | >10000 | 1.00 |
| IDU | MSM | 18.12 | 293.11 | 1.00 |
| HET | MSM | 14.13 | >10000 | 1.00 |
| MSM | HET | 6.45 | >10000 | 1.00 |

*: The transmission relationships with posterior probability ≥0.8 were selected. HET, heterosexual. MSM, men who have sex with men. IDU, injecting drug users.

**Table S10.** BSSVS results of CRF07_BC-O in the Risk-Sex

| From | To | Mean counts | Bayes Factor | Posterior probability^*^ |
| --- | --- | --- | --- | --- |
| IDU-Male | HET-Male | 122.11 | >10000 | 1.00 |
| IDU-Male | HET-Female | 86.83 | >10000 | 1.00 |
| HET-Male | HET-Female | 39.42 | 5926.90 | 1.00 |
| IDU-Male | IDU-Female | 26.05 | >10000 | 1.00 |
| IDU-Male | MSM | 16.51 | >10000 | 1.00 |
| HET-Male | MSM | 11.56 | 443.53 | 0.99 |
| MSM | HET-Male | 5.38 | >10000 | 1.00 |
| HET-Female | MSM | 3.16 | 1479.28 | 1.00 |
| IDU-Female | HET-Male | 0.56 | 306.38 | 0.99 |

*: The transmission relationships with posterior probability ≥0.8 were selected. Risk-Sexs were classified into HET-Female (females in heterosexuals), HET-Male (males in heterosexuals), IDU-Male (males in injecting drug users), and MSM.
